# Applications of massively parallel sequencing in forensic genetics

**DOI:** 10.1590/1678-4685-GMB-2022-0077

**Published:** 2022-09-19

**Authors:** Thássia Mayra Telles Carratto, Vitor Matheus Soares Moraes, Tamara Soledad Frontanilla Recalde, Maria Luiza Guimarães de Oliveira, Celso Teixeira

**Affiliations:** 1 Universidade de São Paulo, Faculdade de Filosofia, Ciências e Letras de Ribeirão Preto, Departamento de Química, Laboratório de Pesquisas Forenses e Genômicas, Ribeirão Preto, SP, Brazil.; 2 Universidade de São Paulo, Faculdade de Medicina de Ribeirão Preto, Departamento de Genética, Ribeirão Preto, SP, Brazil.

**Keywords:** Next-Generation Sequencing (NGS), DNA analysis, RNA analysis, methylation, genetic polymorphisms

## Abstract

Massively parallel sequencing, also referred to as next-generation sequencing, has positively changed DNA analysis, allowing further advances in genetics. Its capability of dealing with low quantity/damaged samples makes it an interesting instrument for forensics. The main advantage of MPS is the possibility of analyzing simultaneously thousands of genetic markers, generating high-resolution data. Its detailed sequence information allowed the discovery of variations in core forensic short tandem repeat loci, as well as the identification of previous unknown polymorphisms. Furthermore, different types of markers can be sequenced in a single run, enabling the emergence of DIP-STRs, SNP-STR haplotypes, and microhaplotypes, which can be very useful in mixture deconvolution cases. In addition, the multiplex analysis of different single nucleotide polymorphisms can provide valuable information about identity, biogeographic ancestry, paternity, or phenotype. DNA methylation patterns, mitochondrial DNA, mRNA, and microRNA profiling can also be analyzed for different purposes, such as age inference, maternal lineage analysis, body-fluid identification, and monozygotic twin discrimination. MPS technology also empowers the study of metagenomics, which analyzes genetic material from a microbial community to obtain information about individual identification, post-mortem interval estimation, geolocation inference, and substrate analysis. This review aims to discuss the main applications of MPS in forensic genetics.

## Introduction

**Figure 1-:**
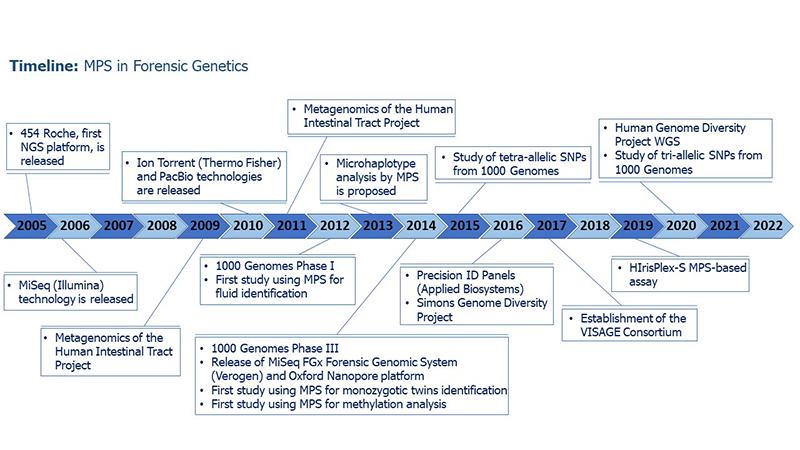
Major advancements in forensic genetics related to MPS use.

The emergence of massively parallel sequencing (MPS) methodology, also known as next-generation sequencing (NGS), represented a revolution for many genomic fields, including forensics. Roche 454 was the first high-throughput sequencing platform launched, in 2005, based on pyrosequencing technique. Since then, the methodology has been considerably improved: while its costs and analysis time have decreased, its sensitivity has raised. New robust instruments have been developed and are commercially available. Although standard DNA analyses in forensic laboratories are made through Short Tandem Repeat (STR) profiling using capillary electrophoresis (CE), this methodology has specific limitations. STR profiling may not be useful when it is needed to distinguish among monozygotic twins, or when the sample recovered is at low quantity/quality, for example. In addition, the CE methodology presents a lower multiplex capability. New approaches are required to extract more information from DNA and help authorities in forensic casework. In this context, MPS presents some important advantages when compared to conventional CE analyses. The main one is the possibility of analyzing thousands of markers, and different types of them, at once. It enables different information to be provided in a single analysis. Another advantage is the capability of dealing with low quantity/degraded samples, which can be very common in forensic casework. Such aspects make MPS a powerful tool for deeper DNA analysis in forensic genetics. Thus, it is not surprising that MPS platforms are being steadily introduced in forensic laboratories. A survey conducted in 2019 among 105 European forensic laboratories showed that 46% of participants already owned an MPS equipment, while 26.7% intended to acquire one within the next two years (Gross et al., 2021). In this review, we aim to discuss MPS methodologies and applications for human forensic genetics. We will address the commercial solutions available in the market, explaining how MPS empowers the analysis of polymorphisms in general and opens up new horizons for forensic genetics and molecular biology (Figure 1).

## Current MPS platforms and panels for forensic applications

Nowadays, Illumina and Thermo Fisher Scientific lead the MPS market. MiSeq and NextSeq benchtop sequencers are Illumina’s top sellers, while the NovaSeq 6000 is considered the most powerful instrument of the company. It is a production-scale sequencer capable of generating 20 billion reads per run within 44 hours, outputting up to 6 Tb of data. 

In 2014, Illumina launched the MiSeq FGx platform, the first developed and validated instrument focused on forensic genomics. Along with it, FGx-specific products were released: the ForenSeq DNA Signature Prep Kit, MiSeq FGx Reagent Kit, and ForenSeq Universal Analysis Software (UAS). In 2017, Verogen, Inc. was established by Illumina, along with Telegraph Hill Partners. Verogen is an independent company that holds the commercial rights to provide Illumina’s sequencing technology to forensic customers. Therefore, Verogen is currently the manufacturer and seller of MiSeq FGx Forensic Genomics System products.

The MiSeq FGx instrument is based on Illumina’s sequencing-by-synthesis (SBS) chemistry. It is designed to analyze single nucleotide polymorphisms (SNPs) and STRs in up to 384 samples per run, depending on the assay, with a maximum read length of 300 nucleotides. It can generate more than 12 million reads, which will be converted later in more than 5 Gb of data. The official website informs that the total overall accuracy of the instrument is above 99.66%, and its Q30 score is higher than 80%. 

The MiSeq FGx platform is recommended for sequencing samples’ libraries prepared with the ForenSeq DNA Signature Prep Kit. This kit includes two DNA primer mixes that allow the amplification of up to 231 markers, with amplicon sizes ranging from 61 to 458bp. The DNA Primer Mix A targets Amelogenin, 27 autosomal STRs, 24 Y-STRs, 7 X-STRs, and 94 identity informative SNPs and has a multiplex capacity of up to 96 samples. The DNA Primer Mix B targets all the markers of kit A plus 24 variants for eye and hair color prediction and 56 biogeographical ancestry SNPs (two of them overlapping), for preparing up to 32 samples. Samples prepared with the ForenSeq kit can also be sequenced on the MiSeq platform. The price per sample for ForenSeq library preparation and MiSeq FGx sequencing is around $50-80, depending on the number of samples per run. 

The ForenSeq UAS was developed for analysis and management of the raw data generated by the MiSeq FGx platform. The software generates FASTQ files, performs alignment, and calls variants from NGS data. Moreover, it visually indicates when a mixture of biological samples is detected.

The MiSeq FGx Forensic Genomics System detects variants within STR sequences and is capable of handling mixtures of biological samples with minor contributors corresponding to less than 5% of the major donor. The system also performs well with degraded DNA samples, since about 80% (191) and 20% (41) of the 231 targeted amplicons are smaller than 200 and 100 nucleotides, respectively. Therefore, the system can be considered a robust and reliable platform for forensic casework (Jäger et al., 2017).

Ion Torrent Personal Genome Machine (PGM) and Ion S5 are Thermo Fisher’s sequencing platforms most used for forensic purposes. They have been available on the market since 2010 and sequencing and analysis procedures can be done in less than three hours. At maximum throughput, this procedure can reach up to 19 hours. Depending on the chosen chip, platforms can generate up to 80 million reads (with length ranging from 200 to 600 nucleotides) that can be converted into up to 15 Gb of data. 

Samples can be prepared for sequencing in Thermo Fisher platforms using several forensic-specific preparation kits from Applied Biosystems. The Precision ID Identity Panel includes 124 SNPs (90 autosomal and 34 in the Y chromosome) for human identification and requires as little as 100pg of DNA. The Precision ID GlobalFiler NGS STR Panel v2 is ideal for deconvolution of sample mixtures and comprises 35 STRs, including the 21 CODIS markers, 9 additional multi-allelic STRs, and 4 sex determination markers. The Precision ID Ancestry Panel includes 165 autosomal SNPs for biogeographical ancestry (BGA) inference. These last two kits require a minimum DNA amount of 125pg. Lastly, the Precision ID mtDNA Control Region Panel targets the 1.2 kb control region of the human mitochondrial genome, requiring only 2pg of DNA. All these kits were optimized for use in degraded DNA samples and the raw data can be analyzed with Thermo Fisher’s Converge Software NGS Data Analysis module.

Another product line manufactured by Applied Biosystems, the Ion AmpliSeq Community Panels for Human Identification, is also compatible with the Precision ID sequencing workflow and comprises five solutions. The first one, the Ion AmpliSeq DNA Phenotyping Panel, was designed in 2015, requires 1 ng of input DNA, and comprises 24 phenotype markers (23 SNPs and one InDel) for eye and hair color prediction (a subset of HIrisPlex-S system addressed in an upcoming section). The AmpliSeq HID Y-SNP Research Panel v1 for paternal lineage identification and paternal biogeographic ancestry inference targets 859 Y-SNPs to infer 640 Y haplogroups. Although it requires 1 ng of input DNA, a validation study has shown that over 87% of the targeted Y-SNPs were called using 100 pg of input DNA, and the right haplogroup was determined down to 25 pg of DNA (Ralf et al., 2019). The Ion AmpliSeq VISAGE-Basic Tool Research Panel includes 153 SNPs for forensic phenotyping and ancestry inferences. Eye, hair and skin color prediction is achieved through 41 SNPs (the HIrisPlex-S system), while biogeographical ancestry inference uses 115 markers (three markers overlap). The average amplicon size of 175 bp makes it useful for degraded samples, and its validation study has demonstrated a robust and reproducible assay, with full profile recovery down to 100 pg of DNA (Xavier et al., 2020). The Ion AmpliSeq PhenoTrivium Panel covers 200 phenotype and ancestryinformative autosomal SNPs (the same 41 markers from the VISAGE Panel for phenotyping, and 163 for ancestry inference) plus 120 lineage-specific Y-SNPs, totalizing 320 SNPs. The mean amplicon lengths are 78 bp and 113 bp for autosomal and Ychromosome targets, respectively, and, although the panel requires 1 ng of input DNA, reliable phenotype and ancestry predictions were obtained down to 25 pg of DNA (Diepenbroek et al., 2020). The last panel is the Ion AmpliSeq MH-74 Plex Research Panel, including 74 microhaplotypes with amplicons ranging from 157 to 325 bp to cover 230 SNPs. This panel is useful for biogeographical ancestry inference, and mixture detection and deconvolution, complementing STR results obtained by CE. Full profiles were detected using down to 25 pg of input DNA (Oldoni et al., 2020). Data generated from all these panels can be analyzed with the same software used for the Precision ID sequencing workflow.

Table 1 summarizes the existing Illumina and Thermo Fisher Scientific panels for forensic applications.

**Table 1-:** MPS panels for forensic applications.

Panel	Manufacturer	Number and types of markers	Chromosomes	Recommended input DNA	Purposes
ForenSeq DNA Signature Prep Kit	Verogen	Mix A: Amelogenin + 58 STRs + 94 SNPs Mix B: mix A + 78 SNPs	Autosomes Y-chromosome X-chromosome	1 ng, with sensitivity as low as 62.5 pg	Human identification Mixture deconvolution Eye and hair color prediction Biogeographical ancestry inference
Precision ID Identity	Applied Biosystems	124 SNPs	Autosomes Y-chromosome	100 pg	Human identification
Precision ID Global Filer NGS STR v2		35 STRs	Autosomes Y-chromosome X-chromosome	125 pg	Human identification Mixture deconvolution
Precision ID Ancestry		165 SNPs	Autosomes	125 pg	Biogeographical ancestry inference
Precision ID mtDNA Control Region		1.2 kb control region	mtDNA	2 pg	Maternal lineage identification Maternal biogeographical ancestry inference
Ion AmpliSeq DNA Phenotyping		23 SNPs + 1 InDel	Autosomes	1 ng	Eye and hair color prediction
Ion AmpliSeq HID Y-SNP Research v1		859 SNPs	Y-chromosome	1 ng, with right haplogroup determination down to 25 pg	Paternal lineage identification Paternal biogeographical ancestry inference
Ion AmpliSeq VISAGE-Basic Tool Research		152 SNPs + 1 InDel	Autosomes	1 ng, with full profile recovery down to 100 pg	Eye, hair and skin color prediction Biogeographical ancestry inference
Ion AmpliSeq PhenoTrivium		319 SNPs + 1 InDel	Autosomes Y-chromosome	1 ng, with reliable predictions for phenotypes down to 25 pg	Eye, hair and skin color prediction Biogeographical ancestry inference Paternal lineage identification Paternal biogeographical ancestry
Ion AmpliSeq MH 74 Plex Research		74 microhaplotypes	Autosomes	1 ng, with sensitivity as low as 50 pg	Mixture deconvolution Biogeographical ancestry inference

## Polymorphic markers for forensic identification purposes

**Figure 2 -:**
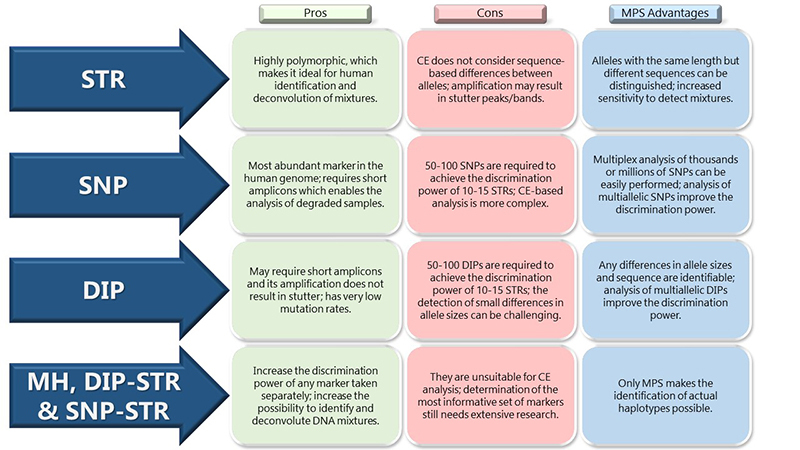
Pros and cons of different sets of polymorphisms and the advantages provided by MPS for their analysis for forensics.

Massively Parallel Sequencing is frequently used to study genes associated with diseases or disorders. However, an increasing number of laboratories have been using this method for forensic genomic analyses too. It brings a lot of advantages because it allows the simultaneous analysis of several regions at the same time, making it possible to complement the information offered by different markers such as STRs, InDels, SNPs and analyze them in a more complete context (Figure 2).

### Short tandem repeats

STRs present a high level of polymorphism, allowing accurate discrimination even between highly related individuals and making it ideal for human differentiation. Capillary electrophoresis (CE) is the most common methodology for STR analysis used in forensic genetics workflow. Allele calling with CE is based on fragment length by comparison with a locus-specific allelic ladder without taking all the structure of the allele into account (Peng et al., 2020). In other words, it does not consider any sequence-based differences between alleles of the same size.

Alleles with the same length but different sequences can be distinguished with MPS technology, which allows a comprehensive analysis of isometric alleles. So, the resulting STR profiles from MPS provide more information than just the number of repeats of a given allele. This technique also offers important advantages for the interpretation of mixed samples. The analysis of nucleotide variation between STR alleles substantially increases allele diversity and discrimination power of a given STR locus, being helpful to deconvolute mixtures, to maximize information retrieved from partial profiles, and to enhance kinship analysis (Pitterl et al., 2010; Bornman et al., 2012).

However, accurate STR genotyping from MPS data has been challenging because of the long lengths of many STR alleles and high sequencing error rates, particularly at the end of sequenced reads. Therefore, high coverages are necessary to obtain reliable genotypes. Some tools were developed for STR analysis from MPS data, such as STRait Razor (Warshauer et al., 2013), toaSTR (Ganschow et al., 2018), and HipSTR (Willems et al., 2017). Many studies have shown successful STR genotype calling from high coverage MPS data using different bioinformatic tools (Valle-Silva et al., 2022). These and other similar papers described some concerns as the high coverage required for accurate genotyping, difficulties to detect longer alleles due to reads of limited sizes, and mutations in flanking regions leading to null alleles (van der Gaag et al., 2016; Tao et al., 2019; Aalbers et al., 2020). Despite the continuous need to improve sequencing technology and bioinformatic tools to enhance STR analysis from MPS data, the countless advantages that current NGS technology offers for STR analysis already endorse its use in forensic casework. 

### Insertion/deletion

Deletion-Insertion Polymorphisms (DIPs), also known as InDels, can be defined as the insertion or deletion of one or many nucleotides in a DNA sequence. They are mostly bi-allelic variations with average allele sizes ranging from one to five bases (Oldoni and Podini, 2019). Nevertheless, in some cases, they can present lengths of up to 10,000 bp (Murthy et al., 2015). They are the second most abundant polymorphisms in the human genome. So far, more than 3.5 million InDels have been identified through massively parallel sequencing (The 1000 Genomes Project Consortium *et al.*, 2015). When compared to STRs, DIPs present lower mutation rates (~2.9 x 10^-9^) (Nachman and Crowell, 2000), and its amplification and sequencing result in shorter amplicons in a procedure that does not result in the production of stutter, which makes it an interesting marker for forensic purposes (Zaumsegel et al., 2013; Al-Snan et al., 2021).

InDels have been successfully used for ancestry inference and human identification. Three panels for ancestry estimation involving major biogeographical groups were developed in the last decade: 48 InDels to evaluate sub-Saharan African, European, and Native American ancestries (Santos et al., 2010), 46 InDels selected to infer ancestry contributions from Africans, Europeans, East Asians and Native Americans (Pereira et al., 2012), and 21 DIPs to differentiate European, African and Asian ancestries (Zaumsegel et al., 2013). Other panels are proper to evaluate ancestry involving recently differentiated groups within a restricted biogeographical area. For example, a panel of 39 InDels was developed and validated to infer ancestry within Chinese populations. Its high combined discrimination power (0.999999999931257) makes it also useful for individual identification and mixture deconvolution (Lan et al., 2019; Zhang et al., 2021b). For Asian populations, there are two additional InDel panels, comprising 12 and 21 markers, respectively (Sun et al., 2016; Zhu et al., 2021). Other InDel panels have been recently developed and validated for mixture interpretation and human identification on degraded samples, proving to be informative, robust, reliable, and sensitive (Jin et al., 2021; Chen L et al., 2021).

For developing all these sets of AIMs, the genotyping procedure was carried out in conventional methods (PCR/CE). Even though, such studies represent important efforts for identifying informative markers to be incorporated in larger MPS assays in the future.

DIPs are usually analyzed along with other polymorphisms, and the MPS methodology simplifies this process. A panel consisting of 1,204 markers (1,176 SNPs and 28 InDels) was developed to interpret DNA mixtures. Thirty-four DNA mixtures (26 nondegraded and eight artificially degraded), with ratios ranging from 1:29 to 1:99, were analyzed through MPS (Illumina HiSeq 2000 sequencer). This panel was capable of accurately identifying the minor contributors in all mixed samples, leading to the conclusion that large panels of bi-allelic markers are sufficient to identify and deconvolute DNA mixtures (Hwa et al., 2018). 

No MPS commercial kits focusing on InDels are available yet. However, this may change soon. As discussed above, this kind of polymorphism has gained a lot of attention in the past decade. In this context, MPS allows a high-resolution identification of the InDel sequence, enabling the identification of other types of polymorphisms in its sequence or in the neighborhood, which can increase its discrimination power. For example, a panel of 68 InDels for human identification was sequenced on the MiSeq platform, using the Nextera Rapid Capture Custom Enrichment Kit (Illumina, Inc.), enabling the identification of surrounding SNPs, composing DIP-SNP microhaplotypes. This finding increased the discrimination power of 22 out of the 68 InDels (Wendt et al., 2016), a welcome feature in forensic genetics.

### Single nucleotide polymorphisms

SNPs consist of single base shifts in DNA sequence. This type of marker is the most common in the human genome, with more than 84 million SNPs identified so far (The 1000 Genomes Project Consortium *et al.*, 2015). They have been widely studied for forensic purposes due to the shorter PCR amplicons and sequencing reads required for amplifying and sequencing such polymorphisms, increasing the odds of a successful analysis in degraded samples (Kayser and de Knijff, 2011). Due to the higher heterozygosity, higher mutation rate (~10^-3^ *vs.* ~10^-8^), and, consequently, increased discrimination power of STRs when compared to SNPs (Wendt and Novroski, 2019), the analysis of 50-100 SNPs is necessary to achieve a discrimination power similar to that obtained with 10-15 STRs (Sanchez et al., 2006; Pakstis et al., 2010). Fortunately, with the use of MPS platforms, the multiplex analysis of a much greater number of SNPs is not a problem anymore and can be easily performed. Larger human MPS-driven projects, such as the 1000 Thousand Genomes Project (The 1000 Genomes Project Consortium *et al.*, 2012; The 1000 Genomes Project Consortium *et al.*, 2015), Human Genome Diversity Project (Bergström et al., 2020), and Simons Diversity Project (Mallick et al., 2016), enabled the annotation of millions of new SNPs, in addition to contributing with worldwide allelic frequency data. This allowed several types of research involving SNPs to be carried out. SNP-based panels for human identification have already been developed, such as the SNP*for*ID 52-plex (Sanchez *et al.*, 2006) and the 92-Plex IISNPs (individual identification SNPs) (Pakstis *et al.*, 2010). The ForenSeq DNA Signature Prep Kit (Illumina) contains 50 and 48 SNPs from SNP*for*ID and IISNPs panels, respectively, while 48 and 46 SNPs are included in the Precision ID Identity Panel (Thermo Fisher), respectively.

Another great contribution of MPS methodology was the annotation of multi-allelic SNPs. Multi-allelic SNPs can also be valuable for encountering mixed DNA patterns, by detecting the presence of a third or fourth allele in a specific site (Phillips et al., 2004). Using the 1000 Genomes Project sequencing data of 2,504 individuals from 26 populations (The 1000 Genomes Project Consortium 2012; The 1000 Genomes Project Consortium 2015), a total of 273,566 multi-allelic SNPs spread across the 22 autosomes and the X chromosome have been annotated so far (Phillips *et al.*, 2020a). The majority of them (271,934) were tri-allelic, while the remaining (1,632) were tetra-allelic SNPs. Multi-allelic SNPs can be amplified with the same efficiency as binary SNPs and present a higher discrimination power, making them useful in the identification of missing persons. After identifying the multi-allelic SNPs present in the genome, the 1,270 most polymorphic tri-allelic SNPs were selected and included in a large-scale MPS multiplex panel for missing person identification, which can be a helpful tool when STR analysis fails (Phillips *et al.*, 2020a). From the set of tetra-allelic SNPs from the 1000 Genomes Phase III, 160 presented high heterozygosity, and the 24 most discriminatory markers were selected. Using this panel, it was possible to detect more than two alleles in at least one marker in more than 99% of cases involving mixtures with African or European donors; for Asians, this number dropped to 92.6% (Phillips *et al.*, 2015). More recently, the MASTiFF (multiple-allele SNP test for forensics) panel was published, comprising 27 tri-allelic and 2 tetra-allelic SNPs. It presents an increased discriminations power in all the five main population groups, with random match probabilities ranging from 10^-15^ down to 10^-20^. In DNA mixture cases, it is estimated that at least one marker will present more than two alleles in 99.8% of cases (Phillips *et al.*, 2020b). Although these studies were based on the SNaPshot methodology, such small panels will contribute to the development of an enlarged panel based on MPS, which will be much more powerful for DNA mixture deconvolution.

### Ancestry and forensic DNA phenotyping markers

In 2007, the SNP*for*ID consortium developed a “single-tube” multiplex assay containing 34 ancestry informative SNPs (AISNPs), which focused on the European, African, and Asian population groups (Phillips et al., 2007; Fondevila et al., 2013). This set was put to test in the 11-M Madrid bomb attack investigation and presented important insights regarding the responsible terrorist group (Phillips *et al.*, 2009). As time passed by, two major sets of AISNPs, composed of 128 (Kosoy et al., 2009) and 55 AISNPs (Kidd et al., 2014) were developed. Both of them used TaqMan arrays and included AIMs that could differentiate Europeans, Amerindians, Africans, Asians (East and South), and Oceanians. Those sets were added to Applied Biosystems’s Precision ID ancestry panel (Thermo Fisher), and together they are even capable of distinguishing North-East, South-West and South-East Asians independently (Lee et al., 2018). Kidd’s set was also added to Verogen’s ForenSeq (Frégeau, 2021).

Another area of study that has gained a lot of attention in the last decade is Forensic DNA Phenotyping (FDP). This technique aims to predict externally visible characteristics (EVCs) of an individual (such as eye color, hair shape, or presence of freckles, for example) analyzing only DNA variants (mainly SNPs). The main idea is that such information could provide additional leads for police when the investigation is stuck (Kayser, 2015). However, EVCs are complex polygenic traits, and hundreds of genes can be involved in its determination. Amongst EVCs, human pigmentation is the first one to have a tested and validated predictive tool. The HIrisPlex-S system is capable of simultaneously predicting eye, hair, and skin color based on 41 markers (40 SNPs and one InDel) (Walsh et al., 2011; Walsh *et al.*, 2013; Chaitanya et al., 2018). MPS assays for Ion Torrent and MiSeq platforms were proposed to obtain HIrisPlex-S genotypes. Full profiles for the 41 markers were obtained from 100pg and 250pg of template DNA, respectively, attesting the high sensitivity of platforms (Breslin et al., 2019). Moreover, the authors encouraged the use of MPS for forensics due to its enlarged multiplex capacity when compared to conventional methods.

Despite HIrisPlex-S being the most established phenotyping tool, its predictive power can be improved, particularly to deal with non-European or admixed populations. Since association studies between genotype-phenotype are performed mostly in European populations, non-European populations may present other associated alleles. We can cite, for example, the *EDAR* gene, associated with straight hair in Asians, and *MFSD12*, associated with skin color in Native Americans/East Asians (Tan et al., 2013; Adhikari et al., 2016; Lona-Durazo et al., 2019; Adhikari *et al.*, 2019). Therefore, it is important to maintain the efforts in identifying new markers associated with physical traits and to conduct studies in different populations to validate the results. Genome-wide association studies (GWAS) are usually performed through array genotyping, which usually lack rarer variants and most of the genetic variability involved in the phenotypes’ emergence. MPS platforms overcome such limitations, with the possibility of performing whole-genome or exome sequencing, enabling the identification of rare variants, or targeted assays, when only desired regions of DNA are sequenced, enabling a deeper study and characterization of a determined gene and its variants. As long as new markers are identified and used for the development or enhancement of predictive models, they can be integrated into commercial panels. Currently, a set of 24 markers from HIrisPlex-S for eye and hair color prediction is included in the ForenSeq DNA Signature Prep Kit and the Ion AmpliSeq DNA Phenotyping Panel. All the HIrisPlex-S’ 41 markers are covered by the Ion AmpliSeq VISAGE-Basic Tool Research Panel and Ion AmpliSeq PhenoTrivium Panel.

In 2017, the VISAGE (Visible Attributes Through Genomics) Consortium was established. It consists of 13 partners from academic, police, and justice institutions of eight European countries and aims to develop integrated predictive tools for appearance, age, and ancestry based on MPS for DNA analysis. Their basic tool accounts for 115 and 41 SNPs for ancestry and appearance (HIrisPlex-S markers for eye, hair, and skin color) prediction, respectively, totalizing 153 SNPs (with three overlapping SNPs). It has been validated on the Ion S5 platform using an AmpliSeq design pipeline and is now available on Ion AmpliSeq VISAGE-Basic Tool Research Panel (Xavier et al., 2020). It has also been validated on the MiSeq FGx System, using the PowerSeq chemistry (Promega) for amplification and library preparation (Palencia-Madrid et al., 2020).

VISAGE’s enhanced tool will use more than 500 markers (autosomal, X, and Y-SNPs and microhaplotypes) to infer ancestry and predict eye, hair, skin, and eyebrow color, hair shape, presence of freckles, and male baldness. It is under validation and not yet publicly available.

### Microhaplotypes

When talking about reliability and exclusion or discrimination power, the first markers that come to mind are STRs, due to their multi-allelic nature and high variability*.* While the same power could be achieved with larger panels of SNPs, forensic human identification still faces difficulties implementing large-scale SNP assays to their labs (Cheung et al., 2019; Standage and Mitchell, 2020; Pang et al., 2020)*.*

Microhaplotypes (or microhaps) may represent a feasible alternative. They consist of three or more genetic markers found nearby, in DNA segments of around 150 to 300 bp, in which the haplotype is made of the allelic combinations observed in the same amplicon. This characteristic makes possible the existence of multiple genotypes even when only bi-allelic SNPs are present (Oldoni et al., 2020; Pang et al., 2020; Yang et al., 2022).

SNP microhaplotypes represent an outstanding alternative to both SNPs and STRs in the contexts of a missing person, relationship and human identification, non-human DNA analysis, sample mixtures, and biogeographical ancestry since a thorough selection of those markers could establish a single MPS assay to deal with all those elements. These markers also avoid problems commonly seen in STR assays, such as stutters peaks/reads, which sometimes complicate the identification and deconvolution of mixtures when the difference between the major and minor DNA donors is high (Oldoni et al., 2019; Standage and Mitchell, 2020).

Microhaps assays are highly sensitive, requiring as little as 50pg (8 cells) of DNA sample, and it could also differentiate mixtures with 2 donors up to a 49:1 ratio and mixtures with up to 6 DNA donors. The number of contributors was accurately defined for 2- to 4-person mixtures in more than 95% of the evaluated samples, and more than 83% accuracy was obtained for 2- to 6-person mixtures (Yang et al., 2022). Usually, when using electrophoretic methods, very little or no information can be gathered from mixtures where less than 5-10% of total DNA belongs to the minor donor. However, the major donor can be always defined and the minor donor could be identified most of the time when using an appropriate number of microhaplotypes. Once the contributors’ proportions are defined in a given DNA mixture, it becomes easier to assign alleles of certain loci to the major and minor contributors (Chen et al., 2019). In fact, Van der Gaag *et al.* demonstrated that this technology allows detecting all alleles of the minor contributors even when they represent as little as a 1% contribution in mixed samples (van der Gaag et al., 2018). In this context, they can be remarkably useful for noninvasive prenatal paternity tests using cell-free fetal DNA (cffDNA). cffDNA is found in very low proportions in the mother’s plasma and has around 100 to 200bp, rarely reaching up to 250bp (Yang *et al.*, 2017; Ou and Qu, 2020; Bai et al., 2020).

Since many SNPs and InDels are already being used as AIMs, it is not surprising that microhaplotypes may represent a huge tool to identify biogeographic ancestry as well. A good set of AIMs organized into microhaps would grant not only ancestry information, but human identifying potential as well (Zhu et al., 2019). It has already been shown that the major worldwide population groups (Africans, South-West Asians and Europeans, East-Asians, Americans, and the Pacific Islanders) can be defined with only 31 microhaplotypes (Oldoni et al., 2019).

In the context of microhaplotypes, two types of compound genetic markers, deletion-insertion polymorphisms amplified with STRs (DIP-STR) and SNPs amplified with STRs (SNP-STR), are getting attention. DIP-STRs identified through MPS assays can be easily genotyped using CE platforms present in every forensic lab. Also, sequence-based information from SNP-STRs and DIP-STRs is of paramount importance to design allele-specific PCR primers (targeting the alternative SNP or DIP alleles), enabling the amplification of alleles from the minor DNA contributor that are absent in the genetic background of the major DNA source, thus eliminating the masking effect of the main DNA contributor (Hall and Castella, 2011; Oldoni et al., 2015).

## DNA methylation for age prediction

In forensics investigations, biological traces left behind in a crime scene may provide additional information about the donor, such as chronological age, becoming a lead that could help narrow down the number of suspects, especially when other information is unavailable. Age-related changes in DNA methylation (mDNA) have been associated with longevity and cellular senescence (Bell et al., 2012). Therefore, it is possible to employ these epigenetic changes for age prediction, an element that falls within the Forensic DNA Phenotyping scope.

DNA methylation is currently one of the most promising age-predictive biomarkers (Hong et al., 2017). DNA methylation corresponds to the attachment of a methyl group (-CH_3_) at the 5’-carbon of a cytosine pyrimidine ring in CpG dinucleotides. Most CpG dinucleotides are methylated, except those located at the CpG islands (CGIs). CpG islands are short-strand regions (05-2.0 kb), with high GC content and a higher frequency of CpG dinucleotides when compared to the average genome, usually located in the 5’ regulatory regions of genes. Conversely, intragenic CGIs are more prone to become methylated, especially during development and differentiation (Deaton et al., 2011). Additionally, methylation in CGIs represses gene expression; thus these regions may show tissue-specific patterns of DNA methylation (Moore et al., 2013).

Methylation patterns are dynamic over time. The machinery responsible for mDNA pattern maintenance accumulates errors over time, leading to an epigenetic drift with aging (Teschendorff et al., 2013). Therefore, these age-specific markers show locus-specific hypermethylation and global hypomethylation (Xiao et al., 2016). Notwithstanding, some regions show methylation patterns that are positively or negatively correlated with the chronological age of an individual. Thus, by measuring the methylation status derived from a DNA sample, it is possible to predict the individual’s age (Horvath, 2013).

In the last decade, many studies have proposed mDNA-based age-prediction models, the majority showing a mean error ranging between 4-8 years (Bocklandt et al., 2011; Hannum et al., 2013; Horvath, 2013). Among the techniques most employed for mDNA analysis are targeted methylation detection using pyrosequencing, microarray-based methods, such as the Illumina Infinium HumanMethylation450 (450K) Beadchip array-based platform, qPCR, and high-resolution melting analysis (Madi et al., 2012; Hamano et al., 2016; Solomon et al., 2018). All these methods require an initial bisulfite treatment step, which converts unmethylated cytosines to uracils, whereas the methylated cytosines remain unaltered (Madi *et al.*, 2012).

In 2014, Weidner et al. (2014) described a protocol using bisulfite pyrosequencing of 151 blood samples. They evaluated the methylation levels at age-related CpGs (AR-CpG) located in the *ITGA2B*, *ASPA*, and *PDE4C* genes, providing a method for age prediction with a mean absolute deviation (MAD) from chronological age of 5 years. Nonetheless, the authors highlighted some association with lifestyle parameters, such as gender, body mass index, and alcohol consumption, which may interfere with the epigenetic drift process (Maegawa et al., 2017), thus impacting these predictions. In a subsequent study, Zbieć-Piekarska et al. (2015) evaluated the methylation status of 7 CpGs in the *ELOVL fatty acid elongase 2* (*ELOVL2*) gene of 427 blood samples by bisulfite pyrosequencing. The final model included two CpG sites in *ELOVL2* and enabled age prediction with *R* ^*2*^ = 0.859 and MAD = 5.03. Despite these results, the authors found an accuracy difference between age groups (younger versus 60-80 years old), thus concluding that *ELOVL2* alone is not adequate for accurate age estimation. Huang et al. (2015) reported a model for age prediction based on four AR-CpGs (*ITGA2B_1*, *NPTX2_4*, *ASPA_1*, and *ITGA2B_2*), identified by bisulfite pyrosequencing and stepwise regression analysis from 89 blood samples. They found that the model explained 82% of the variation in age (*R* ^*2*^ = 0.819 and MAD = 7.87). Also studying an Asian population, Park et al. (2016) identified the methylation status of 3 AR-CpGs: cg16867657 (*ELOVL2*), cg04208403 (*ZNF423*), and cg19283806 (*CCDC102B*). Using bisulfite sequencing from 765 blood samples, they obtained a model that explained 91.08% of the variation in age (*R* ^*2*^ = 0.9543 and MAD = 3.35), with accuracies of 77.30% and 57.30% in groups of people younger and older than 60 years, respectively. 

An NGS approach combined with a machine learning analysis for age prediction in forensic samples was employed for the first time only in 2017 by Vidaki et al. (2017a). The authors used a next-sequencing generation protocol based on Illumina’s MiSeq platform to generate a model for age prediction from blood samples based on the identification of 16 CpG sites. As a result, they obtained an age correlation of 0.86 with a mean absolute error of 7.5 years. These results led to the conclusion that NGS shows excellent potential for age prediction, mainly due to its high sensitivity, multiplexing capabilities, and generation of higher-resolution results.

Another utilization of NGS coupled with machine-learning analysis in forensics refers to the study of Naue et al. (2017). They presented an age prediction tool for whole blood based on MPS using a random forest machine-learning algorithm. Data from 13 age-dependent genes (*DDO*, *ELOVL2*, *F5*, *GRM2*, *HOXC4*, *KLF14*, LDB2, *MEIS1*-AS3, *NKIRAS2*, *RPA2*, *SAMD10*, *TRIM59*, *ZYG11A*) analyzed in a sample of 208 individuals was used for algorithm training and 104 individuals were used for further model evaluation. The cross-validation led to a MAD of 3.2 years, with a root-square error (RMSE) of almost four years, whereas a reduced model employing only the top 4 markers (*ELOVL2*, *F5*, *KLF14*, and *TRIM59*) reached an almost identical performance (MAD of 3.2 years and an RMSE of 4.19 years).

Following this, Aliferi *et al.* employed MPS (MiSeqFGx, Illumina) and multiple machine learning methods to predict age based on the DNA methylation status of 12 previously selected CpG sites (Vidaki et al., 2017a). They used support vector machines with polynomial kernel function to analyze 110 blood samples, obtaining an RMSE of 4.9 years and a mean absolute error (MAE) of 4.1 years (Aliferi *et al.*, 2018).

In 2020, Heidegger and colleagues presented the development and technical evaluation from the VISible Attributes through GEnomics (VISAGE) prototype tool for age estimation that includes 32 CpGs located in five genes *ELOVL2*, *MIR29B2C*, *FHL2*, *TRIM59*, and *KLF14* using MiSeq FGx instrument (Illumina) on blood samples (Heidegger *et al.*, 2020). The age prediction estimator tested on blood samples showed a mean standard deviation of 1.4% across ratios.

Despite being less common, samples other than blood have been employed in DNA methylation-based age prediction since the precision of epigenetic age predictors can be improved by considering the cellular type evaluated. Eipel et al. (2016) evaluated 55 samples from swab buccal by pyrosequencing. Based on three CpGs located within *PDE4C*, *ASPA*, and *ITGA2B* genes, they provided a model showing a correlation between predicted and chronological age of *R* ^*2*^ = 0.91 and MAD = 7.03 years. Aliferi et al. (2018) applied the support vector machines with polynomial kernel function to saliva and semen samples using 12 markers. The age prediction model for saliva achieved a MAE of 7.3 years and RMSE of 11.1 years, while no methylation was detected for any marker in the sperm samples. 

Notwithstanding that, Li et al. (2020) identified the methylation status of two CpG sites (cg06979108 and cg12837463) in 38 semen samples using bisulfite pyrosequencing. The linear regression model explained 86% of the variation in age, with RMSE = 3.94 years and MAD = 2.97 years. Heidegger et al. (2022) described the first age estimation model using MPS technology to evaluate 13 CpG sites from 99 semen samples, with the assay validated by five consortium laboratories of the VISAGE project. Then, the assay performance was tested, yielding a MAE of 5.1 years.

Finally, Woźniak et al. (2021) developed three age prediction statistical models for blood (*n* = 160), buccal swab (*n* = 160), and bones (*n* = 161) using an MPS assay. The VISAGE enhanced tool for epigenetic age estimation developed here was based on eight DNA methylation markers (44 CpGs). Briefly, they obtained a model for blood-based on six CpGs from 6 genes, with a MAE of 3.2 years, a model for buccal cells employing 5 CpGs from 5 genes, with a MAE of 3.7 years, and a model for bones with 6 CpGs from 4 genes, showing a MAE of 3.4 years.

In conclusion, NGS-based age prediction shows essential advantages over other techniques, such as higher sensitivity and the possibility of simultaneous analysis of DNA sequence variation and DNA methylation in a single experiment, justifying the efforts to develop and validate this method in the forensic field.

## Fluid and tissue identification

Current forensic analysis employs STR genotyping to establish the individual’s genetic profile, but it does not determine how the sample was deposited (Hanssen et al., 2017). Additionally, DNA samples are recovered from crime scenes in low amounts, being usually degraded or mixed, hampering the achievement of DNA profiling. Therefore, the determination of which body fluids were placed in a victim or crime scene may help the court reach conclusions regarding the dynamics of a crime (Sijen and Harbison, 2021). 

Body fluids and tissues of forensics interest mainly comprise vaginal fluid, menstrual blood, semen, saliva, sweat, urine, and skin. Traditionally, methods for body fluids identification rely on chemical-based or enzymatic methods; however, these tests lack specificity and are sample-consuming. For instance, immunoenzymatic tests provide many false-positive and false-negative results; chemical tests have low specificity, may destroy the samples, inhibit following analyses, and are not specific for humans (Harbison and Fleming, 2016). DNA and RNA-based technologies are currently being developed in forensics to surpass these issues and include tissue-specific methylation, mRNA and microRNA expression, and microbial analysis. Studies employing these technologies for identification of body fluids and tissues are summarized in Table 2.

**Table 2-:** RNA and DNA-based technologies for fluid and tissue identification.

	Tissue/body fluid identified	NGS Platform	Number of markers	Number of samples	Study
mRNA	blood, semen, vaginal secretion, menstrual blood, and saliva	MiSeq System (Illumina®)	33	232	Hanson et al. 2018
	blood, semen, vaginal secretion, menstrual blood, and saliva	MiSeq System (Illumina®)	33	197	Dørum et al. 2018
	nasal mucosa	MiSeq FGx System (Illumina®)	2	12	Chirnside et al. 2020
	blood, semen, vaginal secretion, menstrual blood, saliva, and skin	MiSeq System (Illumina®)	35	63	Ingold et al. 2020
microRNA	blood, semen, vaginal fluid, menstrual blood, saliva, urine, feces, and sweat	HiSeq System (Illumina®)	6	20	Seashols-Williams et al., 2016
	blood and saliva	Ion PGM™ System (Thermo Fisher Scientific)	25	10	Wang et al. 2016
	blood, semen, vaginal secretion, menstrual blood, saliva, and skin	HiSeq and NextSeq (Illumina®)	9	119	Dørum et al., 2019
DNA methylation	blood, semen, saliva, and epithelial tissue	PyroMark Q24 (Qiagen)	4	42	Madi et al., 2012
	blood, semen, vaginal secretion, and saliva	PSQ HS 96A System (Biotage)	8	80	Park et al., 2014
	blood, seminal fluid, vaginal secretions, and buccal swabs	PyroMark Q24 (Qiagen)	4	89	Alghanim et al., 2020
	saliva	MiSeq FGx System (Illumina®)	18	65	Watanabe et al., 2022
Microbiome profiling	vaginal secretion, saliva and skin	Ion PGM™ System	16S rRNA gene	110	Díez López et al. 2019
	blood, semen, vaginal fluid, menstrual blood, saliva, and skin	MiSeq System (Illumina®)	16S rRNA gene	70	Dobay et al., 2019

### mRNA profiling

Since each cell type exhibits a specific transcriptome, RNA detection enables the identification of body fluid or tissue. Significant advantages of mRNA profiling are the possibility of analyzing different sets of body fluid-specific markers in a multiplex assay, and of co-extracting DNA from the same stain, enabling the obtainment of STR profiles (Haas et al., 2009). 

Although nowadays CE is the gold standard, MPS can analyze mRNA with greater sensitivity (Kulstein et al., 2018). A study comparing the conventional PCR/CE method with the MPS approach found that the number of correctly identified RNA markers by MPS was lower. The authors attribute this to the fact that MPS-based techniques evaluate more markers per body fluid, including those expressed at lower levels, which are admittedly more challenging to assess. Nonetheless, both strategies had similar performances when analyzing high input stains (Salzmann et al., 2021). 

Despite the limitations, MPS technologies are successfully employed in RNA sequencing in forensic analysis. Hanson *et al.* developed a targeted multiplex NGS assay comprising 33 mRNA markers to identify five body fluids (blood, semen, vaginal secretions, menstrual blood, and saliva) and skin. After a blind test, 15 of the 16 samples tested had their body fluids correctly identified (Hanson *et al.*, 2018). These same body fluids/tissues were successfully distinguished (overall prediction = 96.6%) using a probabilistic method that relies on NGS read counts to classify 197 body fluid samples based on the mRNA expression levels of 33 markers (Dørum et al., 2018). More recently, Chirnside et al. (2020) developed an RNA sequencing protocol that identified two candidate nasal mucosa markers, *OPRN* and *BPIFA1*. 

One of the major advances in this field was provided by a study that introduced a set of 35 coding region SNPs (cSNPs) that are present in body fluid-specific mRNA transcripts (Ingold et al., 2020). This set of mRNA SNPs made it possible to identify the type of body fluids and assign such fluids to the respective donors using a single mRNA MPS assay. RNA was extracted from 63 stain samples of blood, semen, saliva, vaginal secretion, menstrual blood, and skin: 12 single-source samples, 36 two-person mixtures containing different fluids, and 15 two-person mixtures containing the same body fluid. The mRNA analysis was capable of identifying the body fluids in the stain. Although the cSNP assay successfully identified the body fluids, the authors concluded that more cSNPs are required to increase discrimination capacity, a simple task even considering the benchtop MPS platforms with the lowest throughput.

It is noteworthy that the inherent instability of mRNA may prevent the detection of these markers in some blots exposed to harsh environments, limiting their use in forensic analysis (Setzer et al., 2008; Watanabe and Akutsu, 2020). 

### microRNA profiling

Unlike mRNAs, microRNAs are highly stable in harsh environmental conditions (Mayes et al., 2019). MicroRNAs correspond to non-coding single-strand molecules, 18-24nt in length, which are post-transcriptional regulators of gene expression by directly binding complementary mRNA target sequences, preventing protein synthesis or inducing degradation (Bartel, 2004). Although most of these molecules are restricted to their cells, some of them, referred to as circulating miRNAs, are found in body fluids, such as saliva and urine (Weber et al., 2010), allowing their use in forensics to identify body fluid origin. Due to their short sizes, high stability, and specificity, they are particularly adequate for mixtures of different fluids or degraded samples. As observed for mRNAs, they can be co-extracted with DNA (van der Meer et al., 2013).

The use of miRNAs in forensic body tissue/fluid identification has been evaluated using microarrays and reverse transcription-quantitative PCR (RT-qPCR) analysis until recently. In 2016, Seashols-Willians *et al.* (2016) published the primer high-throughput microRNA sequencing approach to forensic body fluid identification. They sequenced the entire miRNome from eight body fluids (blood, saliva, urine, feces, semen, vaginal fluid, menstrual blood, and sweat) and observed specific expression patterns of six miRNAs (miR-200b, miR-1246, miR-320c, miR-10b-5p, miR-26b, and miR-891a) that can be used to differentiate blood feces, saliva, urine, semen, and menstrual blood fluids. In another attempt, Wang et al. (2016) employed the Ion Personal Genome Machine (Ion PGM™ System) to identify small-RNA molecules from saliva and blood samples. Although the study was limited to 10 samples from two body fluids, besides confirming many previously described miRNAs for these fluids’ identification, they identified one novel miRNA marker for blood and 15 novel miRNAs for saliva. Dørum et al. (2019) developed a method for identifying six body fluids and tissues (blood, saliva, semen, vaginal secretion, menstrual blood, and skin) from 119 samples using a whole miRNome MPS approach. They evaluated the model’s performance using cross-validation and a public miRNA MPS data set. They observed that the full model (composed of 1,034 markers) and the model with a subset of 100 markers performed equally well, classifying the body fluids with a 90% prediction accuracy. A third model, composed of only nine markers, resulted in a much reduced 65% prediction accuracy, suggesting that future attempts to reduce the number of markers may be a challenging task. Notwithstanding, taken together, these studies suggest that tissue-specific miRNAs provide an alternative framework for body-fluid differentiation, particularly for degraded samples.

### DNA methylation profiling

Nowadays, DNA methylation is the method of choice for body fluid identification due to its high specificity and stability, making it a more reliable tool than RNA or proteins. Different cellular and body fluid types have their specific methylation patterns. Then, these tissue-specific differentially methylated regions can be employed to identify tissues and body fluids types (Lokk et al., 2014). 

Pyrosequencing has been widely used in methylation detection studies to discover differential DNA methylation sites or validate other techniques, mainly microarrays. Madi et al. (2012) employed the method to determine the origin of three body fluids frequently found at crime scenes (blood, saliva, and semen), in addition to epithelial tissue. They identified a set of markers that allowed the determination of the four materials: *C20orf117*, *ZC3H12D*, *BCAS4*, and *FGF7*. The *C20orf117* locus differentiated blood from saliva, epithelial, and sperm cells. At the same time, *ZC3H12D* and *BCAS4* provided the differentiation between sperm and the other cells, showing the potential of differential DNA methylation for body fluid typing.

Park et al. (2014) employed a DNA methylation microarray, the Illumina HumanMethylation 450K bead array, followed by pyrosequencing for results validation, to select CpG sites as suitable DNA methylation markers for body fluid (saliva, blood, semen, and vaginal secretion) discrimination. As a result, they successfully identified eight body fluid-specific DNA methylation markers, two for each one of the four body fluids mentioned above.

In 2020, Alghanim employed pyrosequencing to identify the methylation status of potential markers for body fluid discrimination (*n* = 89), considering samples of buccal swabs, blood, seminal fluid, and vaginal secretion (Alghanim *et al.*, 2020). They found two new loci, *NMUR2* and *UBE2U*, showing different methylation patterns for sperm compared to other tested fluids. Additionally, using the methylation status from 3 CpGs from the intergenic region SA-6, they could separate saliva from the other fluids.

Recently, Watanabe et al. (2022) employed bisulfite sequencing using the MiSeq FGx platform to evaluate the saliva specificity of CpGs from three regions previously selected by microarray analysis. They ended up designing an individual-specific saliva identification assay by analyzing the selected CpGs and SNPs present on the same read. With this assay, it was possible to identify the saliva of a specific person from body-fluid mixtures of known contributors. This CpG-SNP approach can also be applied to other body fluid types, such as blood, semen, and vaginal fluid.

### Microbiome profiling

Since different body regions comprise specific microbiome compositions (Gilbert et al., 2018; Proctor et al., 2019), microbial profiles can also be used as indicators to infer the body origin of specific samples.

The identification of vaginal secretion in a crime scene can support sexual assault. Vaginal microbial composition is relatively stable, with *Lactobacillus spp.* as the main constituent of a healthy vaginal secretion. Therefore, some studies have been employing microbiota-based approaches to identify fluids found in a scene of the crime. Akutsu et al. (2012) developed a PCR-based method to detect 16S rRNA genes of *Lactobacillus spp.* to identify vaginal fluid (*n* = 10). They concluded that 16S rRNA genes of *Lactobacillus crispatus*, *Lactobacillus jensenii*, and *Atopobium vaginae* could be used in a personal identification of vaginal fluid once they are explicitly found in this fluid. Using a similar PCR-base method (*n* = 12), Doi et al. (2014) identified the presence of vaginal secretions by the presence of the Lactobacillus genus. Giampaoli et al. (2012) developed a multiplex-PCR assay (ForFLUID kit) to detect *Lactobacillus spp.*, *Staphylococcus aureus*, *Streptococcus spp.*, which enabled the discrimination of vaginal fluids (*n* = 24) from saliva (*n* = 9), and fecal (*n* = 4) stains. This ForFLUID assay has been tested for efficacy and reliability in identifying vaginal fluids by diverse laboratories, confirming its utility in vaginal fluid identification (Giampaoli *et al.*, 2014).

Saliva is a biological fluid frequently recovered in crime scenes and may indicate the occurrence of biting, kissing, or licking (Gouello et al., 2021). Díez López et al. (2019) developed a novel approach for identifying three different fluid/tissues - skin, vaginal, and oral samples (*n* = 1636) - based on the 16S rRNA gene massively parallel sequencing of 50 taxon and deep learning networks trained with extensive reference data. The employed approach successfully enabled the simultaneous identification of the three different tissue types, with high sensitivity and specificity, as indicated by AUC (Area Under the ROC Curve) of 0.99 for skin, 0.99 for oral, and 1.00 for vaginal secretion (Díez López *et al.*, 2019).

Stability over time for samples (*n* = 46) from six different body sites was tested considering exposition to room temperature for 30 days to assess if the microbial composition remains the same (Dobay et al., 2019). Except for vaginal and menstrual samples, which show an extensive microbiome overlap, Principal Component Analysis (PCA) revealed sample clustering according to body site, even when the samples are exposed to indoor conditions for 30 days. 

Finally, in the absence of DNA content that prevents STR profiling for human identification, the analysis of the penile microbiome transferred to the victim could be used to identify the attacker. Although very little is known about the penile microbiome, with only a few libraries available, the great difference already observed between penile and vaginal samples highlights a future application of MPS-based genital microbiome in forensic casework (Ghemrawi et al., 2021).

### Identification of monozygotic twins

The differentiation of monozygotic (MZ) twins represents a challenge for forensic genetics. As they arise from a single fertilization event, they basically share the same DNA, and, therefore, cannot be distinguished by standard DNA analysis (STR profiling). This limitation becomes important when a MZ twin is involved in a paternity dispute or a criminal case and, thus, must be distinguished from his identical brother. As STR profiling usually fails, alternative methodologies are required to solve the problem. In this context, MPS can bring new approaches or empower the existing ones. Some of the techniques used for MZ twins’ discrimination consist of whole-genome sequencing (WGS), full mitochondrial (mt) DNA sequencing, microRNA (miRNA) profiling, and analysis of methylation patterns.

Although identical twins derive from a single zygote, mutations can occur after the embryo split during early pregnancy, and, thus, such mutations could be used to distinguish between two MZ twins (Rolf and Krawczak, 2021). Despite being rare, these genomic differences may be tracked by a WGS approach. This methodology is especially important for paternity cases, given that the differences may be transmitted to the child. A group at Eurofins Genomics Campus (Germany) sequenced the whole genome of sperm samples from two “identical” twins and a blood sample from the child of one of them, using Illumina HiSeq 2000 technology. The group found five SNPs present in the DNA of the father and the child, but not in the uncle. These genotypes were also confirmed by Sanger sequencing. The mother’s DNA was also analyzed to ensure that the mutations were inherited from the father. They also collected buccal mucosa and blood samples from the twins to check if mutations present in the sperm would appear in other tissues as well. Four of the five mutations were present in the buccal mucosa, and only one was in the blood, suggesting that the former tissue can be suitable for MZ twins’ discrimination (Weber-Lehmann et al., 2014). A recent study of five additional paternity cases involving MZ twins reported that three of them were successfully solved, one presented weak evidence, and the last one remained unsolved (Rolf and Krawczak, 2021). 

Full mtDNA genome sequencing also poses as an alternative for MZ twins discrimination. This extranuclear genome presents some advantages for forensics: it has a) a high substitution rate, presenting variability among closely-related individuals; b) a high number of copies per cell and a circular genome, being more preserved in degraded samples; and c) a small genome size, decreasing the full sequencing costs (Butler, 2012). In addition, MPS allows the analysis of mtDNA with a high coverage depth. Wang et al. (2015) analyzed the full mtGenome of ten pairs of MZ twins using Illumina’s HiSeq 2000 technology. They observed from one to four point heteroplasmies (i.e., the presence of more than one mtDNA type in an individual due to mutations) in all ten sets of twins, demonstrating the efficiency of ultra-deep mtGenome in differentiating identical twins. Opposing results were observed by Li *et al.*: However, no heteroplasmy differences were observed in the analysis of eight pairs of MZ twins (Li *et al.*, 2017). They suggested that Wang’s data contained many potential errors. Data quality, coverage, and contamination issues may represent solid arguments against the reliability of human mitochondrial DNA heteroplasmy from MPS data (Just et al., 2014).

Notwithstanding that, in a case reported in China, a man was arrested for four crimes (three rapes and a murder). Before his final conviction, he had to be distinguished from his twin brother. A WGS assay in Illumina HiSeq X sequencer was performed with both nuclear and mtDNA of the twins, with 30X and 2000X coverages, respectively. WGS was useless in this case since none of the five potential differences passed quality control. It was possible to discriminate between the twins due to a single mtDNA somatic mutation present in the perpetrator and verified in the evidence of two crimes. The genotype was confirmed by amplification refractory mutation system polymerase chain reaction (ARMS-PCR) and then in the biological samples collected from the crimes by allele-specific PCR and amplicon sequencing (Yuan et al., 2020). Moreover, for the two remaining crimes, the innocent twin was excluded as a suspect based on heteroplasmy observed in two additional mtDNA positions: although such heteroplasmies were shared by the two brothers, very different proportions were observed in the semen of the innocent brother (~18%) and the semen retrieved from the evidence of the four cases (< 1%). These elements corroborate the fact that a meticulous analysis of the whole mtGenome may be more suitable than the tedious, costly, and time-consuming process of whole nuclear genome analysis. 

Another promising approach for distinguishing MZ twins is miRNA profiling. MPS is an ideal technique for that analysis since it provides information about expression patterns of the whole miRNome (Fang et al., 2019). Different studies focusing on diseases reported that miRNAs are differentially expressed in MZ twins (Zarrinpar et al., 2016; Wu et al., 2016; Xiang et al., 2017; Uchino et al., 2018). So far, two studies have evaluated the potential of miRNAs in MZ twins’ differentiation. Through MPS, Fang *et al.* (2019) performed genome-wide profiling of miRNAs in blood samples from four pairs of MZ twins. Around 142 to 176 miRNAs were identified for each individual using the Illumina Hiseq 2500 platform, and 10 to 41 miRNAs presented differential expression within the pair of twins. For each pair of twins, the authors also confirmed by real-time PCR the miRNAs with higher differences on expression levels. One miRNA (miR-451a) was found to be differentially expressed within all four pairs of MZ twins (Fang *et al.*, 2019). Another study used microarray to analyze miRNA expression patterns of seven pairs of MZ twins. They found 545 miRNAs with differential expression, 45 of them overlapping those identified by Fang and colleagues*.* Four of these 45 miRNAs (miR-451a, let-7c-5p, miR-151a-3p, and miR-29b-3p) were detected in two or more pairs of MZ twins. Since a large amount of DNA input was required for array analysis, the authors concluded that this technique may not be as useful as MPS for forensic purposes (Xiao et al., 2019). Finally, both studies have used samples from the Han Chinese ethnic group; it is important to mention that caution is required when extrapolating these results from miRNA analysis since miRNA expression patterns may be affected by an individual’s ethnicity (Ma et al., 2015).

The analysis of DNA methylation is the most studied methodology for MZ twins’ discrimination until now. Methylation patterns change throughout an individual’s lifetime. They are tissue-specific and can be influenced by aging, sex, lifestyle, and environmental factors (Jones et al., 2015; van Dongen et al., 2016; Hannon et al., 2018). The first study to report that this epigenetic modification differs between MZ twins was published in 2005 (Fraga et al., 2005), and, since then, many other attempts to discriminate them by methylation analysis have been made. So far, the methodology employed in such studies includes array chips (Park et al., 2017), array followed by qPCR (Vidaki et al., 2017b; Vidaki *et al.*, 2018), PCR-high resolution melting (Marqueta-Gracia et al., 2018), and other techniques (Xu et al., 2015; Du et al., 2015). In all the studies, at least a fraction of MZ twin pairs were able to be discerned. However, there are no established markers for this analysis yet, and further studies are required.

So far, we are not aware of any studies using MPS technology for forensic twin discrimination through methylation analysis. This may be due to its complex data analysis and the infrastructure required (Barros-Silva et al., 2018). However, MPS brings advantages that can empower the study of methylation patterns, enabling the multiplex analysis of lots of methylation sites at a single base resolution. When the need of analyzing a great number of targeted methylation sites arises, the large-scale multiplex ability of MPS, along with its capacity of dealing with low-quality and low-quantity DNA, will make this technique the most suitable for methylation analysis in forensics (Vidaki et al., 2017b; Vidaki *et al.*, 2018).

## Forensic Genealogy

Genealogy has already been used for centuries to trace families back in time. Since large-scale direct-to-consumer (DTC) DNA tests became available, they have been routinely used by genetic genealogists for family history research and unknown parentage research, involving sperm donors, adoptees, and missing persons. The four main groups of DTC testing are FamilyTree DNA, 23andMe, Ancestry.com, and MyHeritage. Investigative genetic genealogy (IGG), also known as forensic genetic genealogy (FGG), is a new and promising tool for individual identification in murders, rapes, and missing person cases, and had already helped generate leads that solved cold cases around the globe (Thomson et al., 2020; Tillmar et al., 2021; Dowdeswell, 2022). Technical and legal considerations must be examined carefully before the method adoption (Scudder et al., 2020).

**Figure 3 -:**
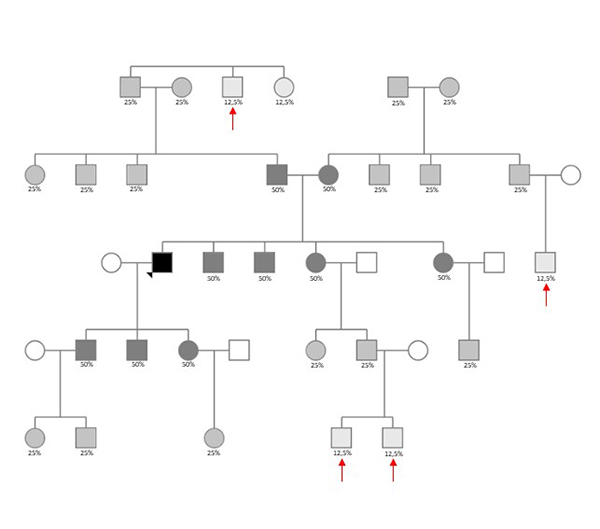
Illustration of a family tree used to solve a crime using Investigative Genetic Genealogy. In this example, after uploading large-scale SNP genotype data from the sample retrieved in the crime scene into GEDmatch, there was a ~12.5% match with a given individual (a DTC consumer). The exploration of different genealogy databases resulted in at least this family tree that included the DTC consumer (represented with a black square). The grey-scale shows individual who share DNA with the DTC consumer, and the numbers bellow each symbol shows the expected average of shared DNA. Red arrows show the four main suspects arising from this genealogy. Additional information from the perpetrator and from these four main suspects, such as gender, age, location (crime scene vs. residence), and predicted ancestry and phenotypes, for example, should be used to narrow down the number of suspects and track the actual perpetrator.

IGG uses hundreds of thousands of SNPs of customized microarrays, such as Illumina OmniExpress or GSA for example, and compare them to public genealogy databases to find segments of shared DNA between an offender and his biological relatives, making it possible to build family trees that include him, which could shorten the investigation (Tillmar et al., 2021). In Figure 3 we present an illustration of IGG application in a putative case, and Table 3 shows the average and the expected range of DNA shared between relatives with different levels of relationship in a family. There are three major requirements to reach a high level of effectiveness for the IGG method: a) large-scale autosomal genotype data available at an affordable price; b) public access to these genotype databases; and c) a well-founded system for analysis (Kling et al., 2021). In this way, WGS data potentially increases the detection power for distant relationships by 5-15% when compared with microarray data (Li *et al.*, 2014).

**Table 3 -:** Average percentage and expected range of DNA shared between family members (modified from a 23andMe table available at https://customercare.23andme.com/hc/en-us/articles/212170668-Average-Percent-DNA-Shared-Between-Relatives).

Relationship	Average % DNA Shared	Range
Identical Twin	100%	-
Parent/Child	50%*	-
Full Sibling	50%	38%-61%
Grandparent/Grandchild, Aunt or Uncle/Niece or Nephew, Half Sibling	25%	17%-34%
1st Cousin, Great-grandparent/Great-grandchild, Great-Uncle or Aunt/ Great Nephew or Niece	12.5%	4%-23%
1st Cousin Once Removed Half 1st Cousin	6.25%	2%-11.5%
2nd Cousin	3.13%	2%-6%
3rd Cousin	0.78%	0%-2.2%

*47.5% for Father-Son relationships

Only one of the four biggest DTC companies working with genetic genealogy, FamilyTree DNA, allows law enforcement agencies to upload genetic information and search for potential relatives. In this scenario, GEDMatch has risen as an invaluable genetic genealogy solution for forensic casework. Established in 2010 and acquired by Verogen in 2019, GEDMatch consists of a global database of autosomal DNA data mostly used for family tree research. It does not sell tests, but accepts DNA tests from other companies and permits the user to upload his profile and search for relatives in its online database for free. Its policy for law enforcement purposes is strict and involves a dedicated website called GED-match Pro. The portal separates police comparisons of GEDmatch data from standard genealogy activities and will also accept data from the ForenSeq Kintelligence Kit, a panel that was announced by Verogen after the acquisition of GEDmatch and consists of the only MPS-based forensic genetic genealogy assay designed specifically for forensic analysis. This assay involves 10,230 SNPs explicitly curated for forensic kinship, and minimizes ethical concerns by excluding medically informative SNPs found in whole-genome sequencing or array-based workflows (Kling et al., 2021). 

It is well known that one of the major barriers that hinder the use of IGG is the difficulty of working with degraded DNA samples (Greytak et al., 2019; Kling et al., 2021). However, this ForenSeq Kintelligence assay requires only 1ng of DNA input per sample and was particularly designed to deal with low-quality samples from hair, bone, teeth, blood, semen, and buccal swabs. It automatically results in a GEDmatch PRO compatible report in less than one hour for investigative lead generation. Although there are no publications concerning the validation and effectiveness of the Kintelligence assay, it is a promising solution developed with detailed bioinformatic analysis regarding different Illumina arrays and the GEDmatch database.

There are indications that there is over a 95% probability of having at least a third cousin match sharing two or more DNA segments in a database with one million samples with ancestry from the same population that originates the query sample. In a matter of fact, GEDmatch had nearly 1 million samples accessible to law enforcement searches in its database, showing that the identification of relatives of Joseph DeAngelo (a.k.a. the Golden State killer) was within expectations (Edge and Coop, 2019).

Greytak et al. (2019) addressed three case studies demonstrating the potential in using IGG to solve cold cases, by using GEDmatch and constructing family trees using census records, vital records, newspaper archives, and social media. In Sweden, Tillmar et al. (2021) also had shown a conclusive lead using IGG, even though the use of this method for Swedish criminal cases is still under evaluation and faces legal, technical, and ethical challenges.

IGG has most of its successful cases centered in the USA and it is gradually starting in other countries, such as Canada, Sweden, and the UK. The majority of cases that benefit from these IGG efforts are murders and /or sexual assaults. In this context, as for the beginning of the year 2022, 44% of the cases involving human remains investigations, where homicide is suspected, were cleared due to the identification of the decedent (Dowdeswell, 2022).

## Metagenomics

Metagenomics studies the collection of genetic material from a microbial community (such as viruses, bacteria, and fungi) living in specific environments (Sleator et al., 2008). Projects describing microbiomes include the Human Microbiome Project Consortium (HMP, https://hmpdacc.org) (NIH HMP Working Group et al., 2009), the Metagenomics of the Human Intestinal Tract Project (MetaHIT, http://www.metahit.eu) (Ehrlich, 2011), and the Canadian Microbiome Initiative (CMI, https://cihr-irsc.gc.ca/).

Before NGS, microbiome samples had to go through culture-dependent methods, which were time-consuming and presented low sensitivity, limiting its use. NGS technologies allowed the production of thousands of genetic information in parallel and decreased the costs for DNA sequences production, establishing a new era in forensics metagenomics (Oliveira and Amorim, 2018). The main approaches in use today correspond to metagenomic shotgun sequencing, which captures all genetic information from a DNA sample, and to amplicon sequencing (e.g., 16S rRNA genes) (Knight et al., 2018).

Nowadays, Illumina MiSeq is the favorite NGS platform in forensic microbiome studies (Díez López et al., 2022). Third-generation sequencing platforms, such as Oxford Nanopore MinION and Pacific Biosciences (PacBio), provide increased resolution and long reads; however, until this moment, these platforms still show significant errors rate, varying between 5-15% (Loit et al., 2019). These new technologies allowed investigating the compositions and dynamics of microbial communities living in diverse environments, increasing microbiome studies in many scientific fields (Knight et al., 2018), including forensic research.

Considering that microorganisms are very abundant in the human body, in the surrounding environment, and crime-related scenes, microbiomes’ analyses could be used as evidence or, at least, complementary information in the investigative process. Among the forensic questions that metagenomics studies can answer are individual identification, post-mortem interval estimation (PMI), and geolocation inference.

### Individual identification

Pieces of evidence suggest that personal identification could be based on a microbiome profile, like a “fingerprint” that differentiates each individual. Franzosa *et al.* evaluated 120 individuals from the HMP project to assess whether the intraindividual variation on the microbiome is sufficient to distinguish individuals (Franzosa *et al.*, 2015). They found gene-level metagenomic codes (i.e., sets of microbes) that discriminate between a person among hundreds of individuals. About 30% of codes from a specific body site uniquely identified an individual up to 10 months later, while gut microbiomes pinpointed 80% of individuals with exceptional stability over time. Another study (Schmedes et al., 2017), using the presence/absence of *Propionibacterium acnes* and the nucleotide diversity of clade-specific markers, described an approach that attributes skin microbiomes to their donors with a high degree of accuracy. Microbiome samples (*n =* 12) from different body sites (*n =* 14), collected over time (>2.5 years), allowed identifying stable clade-specific markers with classification accuracies of up to 100% for cheek, inguinal crease, and popliteal fossa. 

Following this initial work, Schmedes et al. (2018) proposed the hidSkinPlex for human identification, a targeted sequencing method using 286 microbiome markers that target 22 bacterial and phage clades. They obtained accuracies as high as 94% in classifying 24 samples from 8 individuals regardless of the body site origin (i.e, hand, foot, or manubrium), and up to 86% accuracy in predicting body site origin (Schmedes *et al.*, 2018). Woerner et al. (2019) evaluated the hidSkinPlex in samples from the three previously studied body sites in 51 new individuals, reaching 100% individual classification accuracy when conditioning the estimates to a maximum nearest neighbor distance for diversity. Watanabe et al. (2018) described a method using minor taxa of the skin microbiome for personal identification, evaluating 66 samples collected from 11 individuals over two years. Their method enabled classifying individuals with 85% accuracy in this two-year timeframe. Furthermore, they employed the same approach to 837 publicly available skin microbiome samples from 89 individuals to assess the method’s effectiveness, which allowed identifying individuals with 78% accuracy (Watanabe *et al.*, 2018).

Studies using samples other than skin are yet scarce. Tridico et al. (2014) employed human scalp and pubic hairs (*n =* 42) from seven individuals at three time points over 5 months to identify the microbiome associated with them. While the microbiota of male pubic hairs could be readily distinguished from female pubic hairs, scalp hair microbiota showed no correlation with the sex of the donor. Notwithstanding, the authors identified the contributor for the samples and discriminated between the two origins of hair. Additionally, the study revealed that sexually active couples and cohabiting individuals interchange microbiomes during sexual intercourse (Tridico *et al.*, 2014).

Individual identification can also be performed indirectly, associating the microbiome profile retrieved from a touched object or place with that obtained from a suspect, particularly in the absence of touch DNA for obtaining human STR profiles. Studies show that skin-associated bacterial communities persist on surfaces (for instance, computers and mobile phones) for long periods and may be transferred upon touching. Fierer *et al.* described the potential to use bacteria to link touched samples to a specific individual. The authors compared the bacterial communities found on personal computer keyboards to those on the fingers of the keyboard owners (*n =* 3) using a pyrosequencing approach. Moreover, by comparing bacteria left on the personal computer mice (*n =* 9) against a database containing information about 270 hand surfaces (including the owner’s), they linked objects to a specific individual, concluding that bacteria found on a personal object is more similar to the owner´s microbiome than to that of the general population (Fierer *et al.*, 2010).

Another study collected three samples from each participant (*n =* 17) to establish operational taxonomic units from the 16S rRNA gene to quantify the shared bacterial communities among mobile phones and fingers of their owners using Illumina sequencing technology (Meadow et al., 2014). They found that 22% from the overall bacterial community present on a sampled finger were also present on the respective phone, while only 17% of these bacteria were shared with phones from other people. Considering only the most predominant taxa (i.e., those representing more than 0.1% of the sequences in an individual’s dataset), 82% of them were shared between the index fingers of an individual and their own phones.

Lax et al. (2015) also evaluated the microbiome of smartphones using 16S rRNA Illumina sequencing by two different approaches: a) a longitudinal one for assessing the bacterial communities along time, when the mobile phones of two individuals were sampled 24 times (every hour in 12 h cycles on consecutive days), and b) a biogeographic characterization of the microbial communities deposited on the phone surfaces from participants of three different scientific conferences (*n =* 89). The authors found a characteristic bacterial community based on the owner of the material, allowing them to infer the person’s identity based on this associated microbial community (Lax *et al.*, 2015). Yang et al. (2019) also used Illumina sequencing to genotype *Cutibacterium acnes* 16S rRNA in samples from the hands of 10 participants and their possessions (smartphone screen and laptop’s keyboard and touchpad), which allowed the prediction of the owner of an object with 90% accuracy.

Procopio et al. (2021) evaluated the transferability and persistence of the touch microbiome on a glass surface after the deposition of the fingerprint and its exposure for 30 days at room temperature. Both human and microbial DNA was isolated and quantified. While human STR profiles were successfully obtained for only 5 out of 22 “touch DNA” samples, the Illumina sequence-based analysis of the 16S rRNA gene resulted in microbiome profiles for 20 out of 22 “touch microbiome” samples. These results clearly show that the integration of the microbiome analysis together with STR typing is an alternative when low amounts of DNA or degraded samples are available preventing the retrieval of complete human STR profiles (Procopio *et al.*, 2021).

Overall, although promising, the level of accuracy of these microbiome “fingerprints” is yet inadequate for forensic applications. Improvements in statistical methods, in model’s specificity/sensitivity, and in determining the microbial diversity variation across body sites and time, besides the availability of public biobanks describing a “core microbiome” among humans, are necessary for their actual effectiveness in forensic situations (Caenazzo and Tozzo, 2021). 

### Postmortem interval (PMI)

As for postmortem interval (PMI), different taxonomic signatures can be defined for the microbiota from the gut, skin, and bones of the victim during decomposition (Clarke et al., 2017), as well as soil microbiome in each stage of decomposition, since the aerobic reactions occur until oxygen depletion and then anaerobic reactions increase. Thus, different bacteria, fungi, protists, and other microorganisms could be found in each stage of decay (Finley et al., 2015).

The Human Postmortem Microbiome Project (HPMP) gathers scientists around the world to define the human thanatomicrobiome (the microorganisms in internal organs and in blood samples collected after a human dies), the epinecrotic communities (from epithelial tissues, body cavities, and the alimentary canal), the necrobiome (micro and macro-organisms related to decomposition), and the soil microbial communities found in association with body decomposition. They aim at unifying the global research communities and defining an experimental modus-operandi around sampling, processing, sequencing, and bioinformatic analysis of postmortem microbiota (Javan and Finley, 2018).

Different PMI methods are already used in the forensic routine, such as entomology and the stages of the human body decomposition, but the use of thanatomicrobiome and epinecrobiome-based estimation of PMI brings the potential for improving accuracy (Dash and Das, 2020; Roy et al., 2021). The obtained accuracy in PMI prediction for both open-air and buried human remains are really close, being able to predict within 1.82 ± 0.33 days of mean error in a 60-day period of decomposition (Zhang et al., 2021a). Hu et al. (2021) identified that the appendix had a nice microorganism succession rate for human decomposition, and within a 192-hour period the mean error was of 25.79 ± 0.43 hours for PMI estimation. Cartozzo et al. (2021), even demonstrated the promising possibility of defining PMI for submerse samples, using *Sus scrofa* bones in a freshwater river. It is noteworthy that, although these MPS-based studies represent only some initial steps towards the implementation of metagenomics as a forensic tool for PMI estimation, preliminary results are promising and indicate that additional research could lead to more accurate and precise estimates for variable environmental situations.

### Geolocation inference

Inorganic compounds found in the soil are broadly used in forensic investigation, although it has a low potential when we are talking about the differentiation between samples. Palynology has been given a lot of attention, but there is an alternative: the analysis of the soil’s microbiome using metagenomics, which can be used to accurately define the origin of the sample (Khodakova et al., 2013; Demanèche et al., 2017). The touch microbiome could be considered a potentially informative forensic marker for geolocation. Soil has the largest phylogenetic and functional diversity per volume (10^4^ to 10^7^ per gram). Thus, NGS can be used as an objective method to individualize soil samples (Karadayı, 2021). Demanèche *et al.*, demonstrated great potential using a mock test, where they could associate samples with different sites with high definition (Demanèche *et al.*, 2017). 

In the previously described study in which Lax et al. (2015) evaluated the microbiome of smartphones using 16S rRNA Illumina sequencing, shoe samples of the same individuals were evaluated to verify the correlation between the microbial communities from an individual´s shoes with the floor microbiome associated with the site where they were walking. The same two approaches were conducted: a) a longitudinal one for assessing the bacterial communities along time, when the shoes of two individuals were sampled 24 times (every hour in 12 h cycles on consecutive days), and b) a biogeographic characterization of the microbial communities deposited on the shoes sole from participants of three different scientific conferences (*n =* 89). The first approach revealed that bacterial taxa associated with the floor of a particular location often increased in abundance on the shoe soles of a subject while walking through that space. In addition, the second approach enabled (significantly better than expected by chance) the determination of the conference of origin of a given sample, suggesting that different sites maintain a significantly different floor microbial community, which in turn shapes the microbial community associated with the shoes (Lax *et al.*, 2015). 

Although more challenging, understanding the metagenomic map of a city could help with disease surveillance, bioterrorism threat mitigation, health management, geolocation, and human identification. Afshinnekoo et al. (2015) had shown the feasibility of identifying microbiota from New York City public parks and from a hurricane-flooded subway station, although half of their high-quality sequence Illumina reads did not match any known organism, which may mean that it likely represents un-culturable species. The fact of identifying clade-specific samples for different city regions shows the huge potential this analysis could bring to society. However, improvements aiming at the faster characterization of the dynamics of urban metagenomes are of paramount importance for forensic purposes.

Soil microbiome community structures are greatly influenced by soil types, but since it is very unlikely that the soil sample is collected immediately after the crime occurs, it is possible that time and weather can exert a strong influence on the microbiome, which could introduce some bias to the analysis (Karadayı, 2021). Given that, it is possible to foresee a future in which the physical chemistry methods available to soil analysis will be considered together with metagenomics to provide geolocation of a query sample. However, there is no defined protocol establishing the methods of DNA extraction among microbiologists, and since each soil has its own properties, different methods must be validated (Díez López et al., 2022).

In general, irrespective of the application, metagenomics analyses are full of biases (Díez López et al., 2022). Firstly, it is crucial to identify the taxonomic resolution level during analysis. For many species, strain-level identification can be challenging given that very few (if any) genomes from these species are well-represented in genome databases (Nayfach and Pollard, 2016). Furthermore, since metagenomics is based on data comparison, meaningful conclusions require precise quantification of taxa and gene abundances to compare microbiome diversity across studies, taking into account inter and intra-individual variations of the human microbiome, or variation in environmental samples. Further validation of methods and enhancement of reproducibility is of utmost importance (Schmedes et al., 2017; Schmedes *et al.*, 2018; Watanabe et al., 2018; Woerner et al., 2019; Yang et al., 2019; Schmedes *et al.*, 2020).

It is important to take notice that contamination is probably the worst problem surrounding metagenomics analysis. Swabs, envelopes, desks, lab surfaces, tips, and plastic tubes, technicians, and researchers may consist of probable sources of contamination (Neckovic et al., 2021). Apart from the lab issues, metagenomics needs to develop likelihood ratios for forensic purposes to be accepted in court (Díez López et al., 2022). 

Notwithstanding that, metagenomics and microbiome analysis are still at the beginning of a journey, but it has the potential to become a powerful tool for all forensic labs.

## Conclusions

Massively parallel sequencing is a valuable technology that offers advantages that overcome many limitations of standard techniques. Although such techniques are still the most used nowadays, MPS brings different approaches for already known problems and also enables the emergence of new areas of study within forensics. It is expected that MPS cost-benefit will increase over time, and, therefore, it will be more accessible in forensic laboratories, and, thus, more incorporated into the forensic routine. We hope that in the near future, large panels with thousands of markers for forensic use on MPS platforms will be developed, generating different information about an individual in a single analysis. As this technology advances, forensic genetics will advance along with it. Many applications covered in this paper are still in the early stages, but in the next few years, we probably will see these techniques becoming more robust, standardized, and validated, which is mandatory for use in real forensic cases. 
